# Effect of changes in the hearing aid subsidy on the prevalence of hearing loss in South Korea

**DOI:** 10.3389/fneur.2023.1215494

**Published:** 2023-09-12

**Authors:** Chul Young Yoon, Junhun Lee, Tae Hoon Kong, Young Joon Seo

**Affiliations:** ^1^Research Institute of Hearing Enhancement, Yonsei University Wonju College of Medicine, Wonju-si, Gangwon-do, Republic of Korea; ^2^Department of Biostatistics, Yonsei University Wonju College of Medicine, Wonju-si, Gangwon-do, Republic of Korea; ^3^Department of Otorhinolaryngology, Yonsei University Wonju College of Medicine, Wonju-si, Gangwon-do, Republic of Korea

**Keywords:** hearing loss, big-data, hearing aid, hearing disability, prevalence

## Abstract

**Objectives:**

South Korea's National Health Insurance has provided hearing aids to registered individuals with hearing disabilities since 1989. In 2015, hearing aid subsidies increased to approximately US$1,000. This study aimed to understand hearing loss categories in Korea by analyzing patients between 2010 and 2020 and the effect of the 2015 hearing aid policy change on the prevalence of hearing loss.

**Methods:**

The participants were patients registered on the National Health Insurance Service database from 2010 to 2020 with hearing loss. A total of 5,784,429 patients were included in this study. Hearing loss was classified into conductive, sensorineural, and other categories. Patients with hearing loss were classified according to the International Classification of Diseases diagnostic code. Disability diagnosis and hearing aid prescription were defined using the National Health Insurance Disability and Hearing Aid Code.

**Results:**

The increase in hearing aid prescriptions and hearing disability registrations following the subsidy increase impacts hearing loss prevalence. Hearing aid prescription and hearing disability were found to have an effect on increasing hearing loss prevalence in univariate and multivariate analyses. The *r*-value of each analysis exceeded 0.95. Other hearing losses increased rapidly after the increased subsidy.

**Conclusion:**

A hearing-impaired individual must be diagnosed with a hearing disability and prescribed a hearing aid to receive the subsidy. The prevalence of hearing loss was affected by increased hearing disabilities following changes in the hearing aid subsidy and the number of people prescribed hearing aids. Therefore, caution should be exercised when studying hearing loss prevalence over mid-long-term periods.

## Introduction

The burden of hearing loss is substantial at an individual level and over a lifetime. This burden can be exacerbated by negative social attitudes and prejudices toward those who are affected (1). Hearing loss negatively affects communication, psychosocial wellbeing, quality of life, and economic independence (2, 3). Hearing loss during early childhood (5–7 years old) interferes with communication and language development (4). Adults often experience social isolation, stigma, abuse, mental disorders, depression, difficulties with family relationships, limited career choices, job stress, and relatively low income due to this disability (1). Hearing loss can have various causes, degrees, and sites of occurrence. Sudden sensorineural hearing loss is diagnosed within 12 months of onset and usually improves with appropriate treatment (5). An accurate diagnosis of hearing loss is necessary to predict the appropriate treatment and prognosis and rehabilitate the disability (6, 7). Hearing aids are one of the treatment devices used for the rehabilitation of hearing loss. Hearing aid users can potentially improve their physical, emotional, and social quality of life. They are more active than those who do not use hearing aids and suffer from fewer negative effects, such as depression and anxiety (8). The sooner individuals become aware of hearing loss and use hearing aids, the sooner their communication problems will improve (9).

This study used data from the National Health Insurance Service (NHIS). The NHIS uses the National Health Insurance (NHI) to lessen the burden of high medical expenses on the public. The public pays insurance premiums to mitigate the risk of high medical expenses due to illness or injury. The insurance is managed and operated by the NHIS through social consensus. The NHI provides health insurance benefits to the public, meaning that risk is shared and everyone has access to necessary medical services. Data from National Health Insurance subscribers are accumulated in the NHIS database (NHIS DB). Information such as medical history, drug prescription history, and health evaluation is limited to researchers only (10, 11). Hearing aid subsidies are welfare payments paid by the government to patients who need hearing aids to encourage them to purchase hearing aids. South Korea's NHI has provided hearing aids to registered hearing disabilities people since 1989, but it was not enough. The expanded hearing aid subsidy program began in 1997 with partial reimbursement of hearing aid costs (approximately $200). This increased to approximately $250 in 2005 and $1,000 in 2015, with $1,000 being the cost of an entry-level hearing aid (12). To receive a hearing aid subsidy in South Korea, you must be registered as hearing-disabled and receive a hearing aid prescription from an otolaryngologist. Hearing disability registration requires a hearing disability diagnosis. This is determined in a hospital by the results of pure tone audiometry (three or more times), word recognition scores, and auditory brainstem response with an otolaryngologist. Hearing disability in South Korea requires the issuance of a hearing loss certificate from an otolaryngologist; two government-affiliated otolaryngologists review the certificate (12). A study that presented the adequacy of the hearing aid subsidy before the 2015 increase indicated the need for a significant increase in the amount and a follow-up support system for hearing aid users. However, the changes in 2015 focused only on significant increases in hearing aid subsidies (13). The hearing aid subsidy is for any age, and there is an additional payment amount for children who are hearing disabilities people.

A 2015 study analyzing the prevalence of hearing loss and hearing aids in older adults (14) and a study on unilateral hearing loss and hearing aid use in 2020 (15) indicated that the penetration of hearing aids in South Korea was insufficient. However, these studies used data from the National Health and Nutrition Examination Surveys from 2010 to 2012, which did not reflect South Korea's hearing aid subsidy increase in 2015. Previous studies on the hearing aid support system and subsidy increase have discussed the effects of the subsidy and the change in perception of the support system. However, the effect of the hearing aid subsidy on the prevalence of hearing loss was not confirmed (16). Investigating the prevalence of hearing loss can confirm the overall status (treatment rate, diagnosis rate, incidence, recurrence rate, rehabilitation rate, etc.) of patients with hearing loss. Furthermore, examining the current adoption of hearing aids can confirm the effectiveness of South Korea's hearing aid supply policy.

This study aimed to analyze the NHIS DB for patients treated for hearing loss by year, sex, and age and identify domestic hearing loss by type from 2010 to 2020. Moreover, the study assessed the current status of hearing aids for patients with hearing loss.

## Materials and methods

### Participants

South Korea's National Health Insurance covers 98% of the population. The citizens are covered for medical expenses whenever they visit a hospital (10). The participants were patients registered on the NHIS DB and diagnosed with hearing loss between 2010 and 2020. Each patient had a history of hearing loss in the primary or secondary diagnosis and personal information such as year of birth and sex. Additional information was confirmed, such as medical examination and disability diagnosis history. A total of 5,784,429 patients were included in this study. Subsequently, the number of patients for each category was converted to the number of patients per 1,00,000, and the background population was the total population of South Korea in each given year.

Changes in patients with hearing loss by year were analyzed for changes compared to other age groups by comparing increases and decreases between the relevant age group and other age groups. Age is an important variable, and it was thought that the influence would be different depending on the type of hearing loss, so an adjustment was not performed, but an adjustment was made according to the population of each statistical group. Statistical analyses were performed using SAS, version 9.4 (SAS Institute Inc., Cary, NC, USA).

### Definition

**Figure 1:**
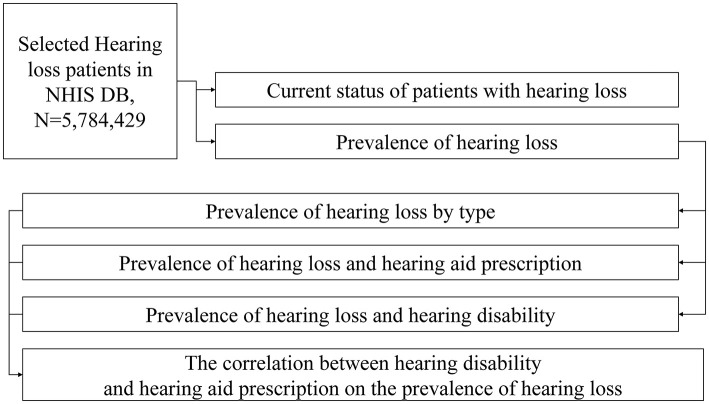
Flowchart of the article.

The data for this study were collected from the NHIS DB. The data were used to classify patients according to their category of hearing loss; the number of patients with hearing loss and disability diagnosis over 10 years was investigated (Figure 1). The types of hearing loss were classified as conductive, sensorineural, or other hearing loss. Sensorineural hearing loss was subdivided into sensorineural, mixed, ototoxic, presbycusis, sudden, and noise-induced hearing loss. This study was approved by the Institutional Review Board (IRB) of Yonsei University Wonju Severance Christian Hospital (CR321338).

The NHIS DB collects the following historical information: medical treatment, drug prescriptions, and health checkups of health insurance-registered individuals. After de-identifying the participant data, the dataset was provided for policy and academic research purposes. National health information data can be provided to health and academic medical fields (associations, universities, research institutes, etc.) for systematic research activities related to public health. Patients with hearing loss were classified according to the International Classification of Diseases, 10th Revision (ICD-10) diagnostic code (major/minor diagnosis) based on the operational definition for each category (Table 1). The respective disability was defined by a disability code after the initial hearing loss diagnosis. In the case of hearing aids, a hearing aid code was defined. We only included the patient's first diagnosis in the DB and did not consider recurrence. However, even with a diagnosis of hearing loss, we cannot be certain that everyone has had their hearing tested. Therefore, the prevalence in our study is an estimate.

**Table 1:** IDC-10 codes by types of hearing loss.

**Types of hearing loss**		IDC-10^*^
Conductive hearing loss		H90.0
		H90.1
		H90.2
Sensorineural hearing loss	Sensorineural	H90.3
		H90.4
		H90.5
	Mixed	H90.6
		H90.7
		H90.8
	Ototoxicity	H91.0
	Presbycusis	H91.1
	Sudden	H91.2
	Noise-induced	H83.3
Other hearing loss		H91.3
		H91.8
		H91.9

* International Classification of Diseases, 10th Revision.

## Results

### General characteristics of patients with hearing loss

**Figure 2:**
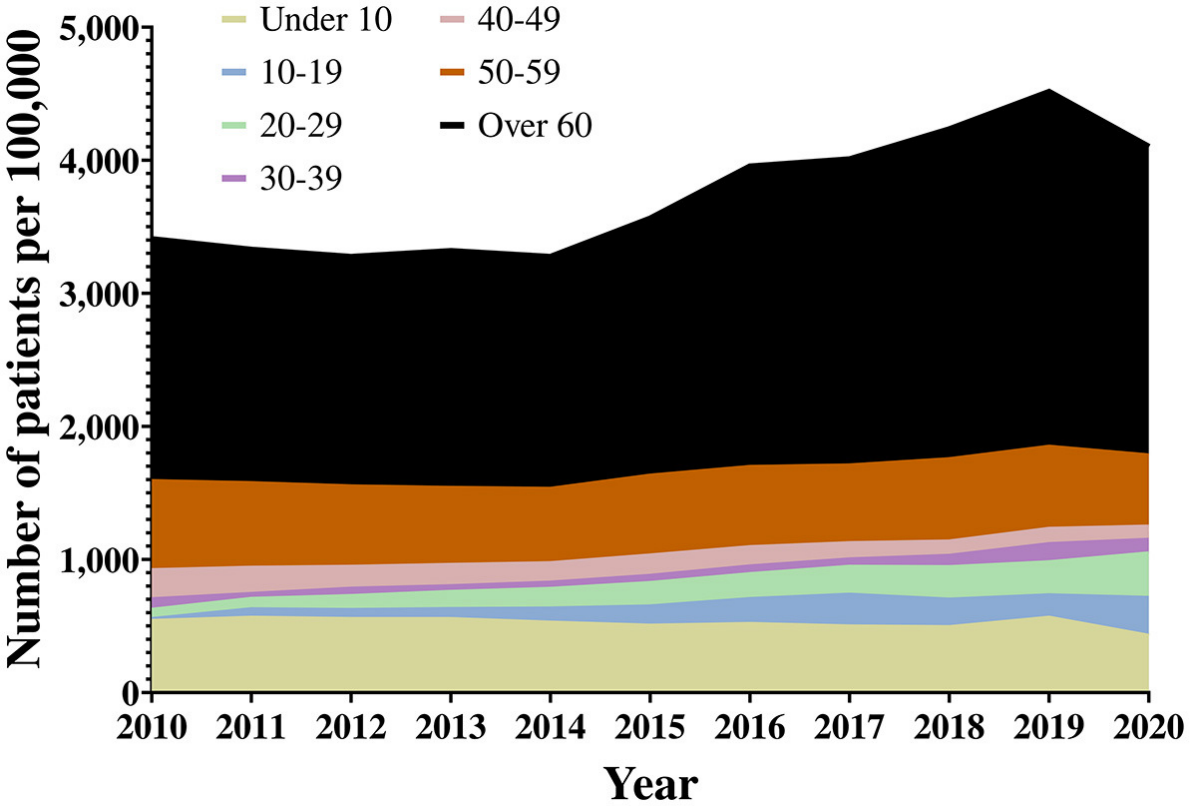
Age distribution of hearing loss patients.

A total of 5,784,429 patients were included in this study. The distribution of hearing loss patients by age ranged from 25,553 patients under the age of 10 years in 2010 to 4,89,419 patients over 60 years in 2020 (Figure 2, Supplementary material). The number of patients with hearing loss per year increased from 6,04,702 in 2010 to 9,42,764 in 2020. From 2014, the number of patients in their 60s also increased significantly. In addition, it can be seen that the number of hearing loss patients in their 20s increased substantially between 2010 and 2020. The number of patients with hearing loss increased substantially from 2010 to 2019 before dropping in 2020. This was likely due to lockdown restrictions, social distancing policies, and an overloaded medical system due to the Coronavirus disease-19 (COVID-19) pandemic (17, 18).

Looking at the compound annual growth rate (CAGR) of hearing loss patients from 2010 to 2020, it is evident that the rate of increase is high among those in their 20s and 30s, and the incidence rate of those under 10 years has decreased significantly. However, prevalence increased in all age groups except those under 10 years. This is not a phenomenon whereby the number of patients with hearing loss increased in any age group because of a single event rather the influence of hearing loss increased in all age groups (Table 2). The hearing aid supply policy changed in 2015. When the CAGR is calculated for 2015, patients in their 20s to 60s showed a more significant rate of increase than in previous years. However, there is a decrease in the under 10s and teenage age groups. Except for 2020, due to COVID-19, the rate of increase is amplified in the under 10s, 50s, 60s, and older age groups. We can also see that the teenage group returns to pre-2015 levels.

**Table 2:** Compound annual growth rate by age and types of hearing loss patients.

**Type of hearing loss**	**Range**	**Under 10**	**10–19**	**20–29**	**30–39**	**40–49**	**50–59**	**Over 60**
All hearing loss	2010–2014	−0.269	1.522	2.566	1.858	0.628	−0.425	−0.443
	2015–2020	−1.843	1.053	2.720	3.063	2.171	1.002	1.579
	2015–2019	1.267	1.366	1.957	2.726	2.021	1.415	2.667
Conductive	2010–2014	−2.316	−0.748	1.693	0.590	−0.341	−0.575	−1.361
	2015–2020	−1.404	−0.679	0.497	1.108	0.577	0.852	1.904
	2015–2019	3.365	0.887	0.763	1.518	0.961	1.871	2.719
Sensorineural	2010–2014	−0.086	1.626	2.417	1.425	0.189	−0.851	−0.404
	2015–2020	−1.395	1.105	2.814	3.470	2.059	0.766	1.698
	2015–2019	1.072	1.190	2.081	3.129	1.905	1.234	2.931
Mixed	2010–2014	3.821	3.784	4.298	3.893	1.979	1.165	0.903
	2015–2020	−7.426	−1.195	1.001	1.618	0.844	−0.519	0.220
	2015–2019	−3.665	−0.454	0.643	1.115	0.558	−0.229	0.849
Ototoxicity	2010–2014	−17.601	−8.108	−9.275	−11.338	−8.473	−10.722	−8.349
	2015–2020	−12.260	−4.735	−11.130	−17.096	−9.497	−6.998	−9.664
	2015–2019	−2.592	−6.706	−11.141	−6.621	−6.873	−1.540	−7.161
Presbycusis	2010–2014	0	0	0	0	−2.159	−2.523	−2.338
	2015–2020	0	0	0	0	−2.114	−3.989	−1.352
	2015–2019	0	0	0	0	−0.934	−1.496	0.798
Sudden	2010–2014	1.634	2.274	3.111	2.326	2.059	1.301	0.774
	2015–2020	−1.783	1.130	3.387	3.356	2.815	1.513	1.326
	2015–2019	1.562	0.278	2.028	2.370	2.549	1.700	1.787
Noise-induced	2010–2014	2.843	0.268	−0.887	−2.481	−1.479	−0.814	0.901
	2015–2020	−10.664	−4.717	−5.297	−3.957	−3.211	−2.058	2.999
	2015–2019	−2.191	−2.271	−3.855	−2.194	−2.144	−1.539	2.341
Other	2010–2014	−0.778	1.369	3.045	3.232	1.216	−0.405	−0.655
	2015–2020	−0.947	2.644	4.196	4.150	3.835	2.749	3.664
	2015–2019	1.942	3.190	3.210	3.883	3.585	3.003	4.672

**Figure 3:**
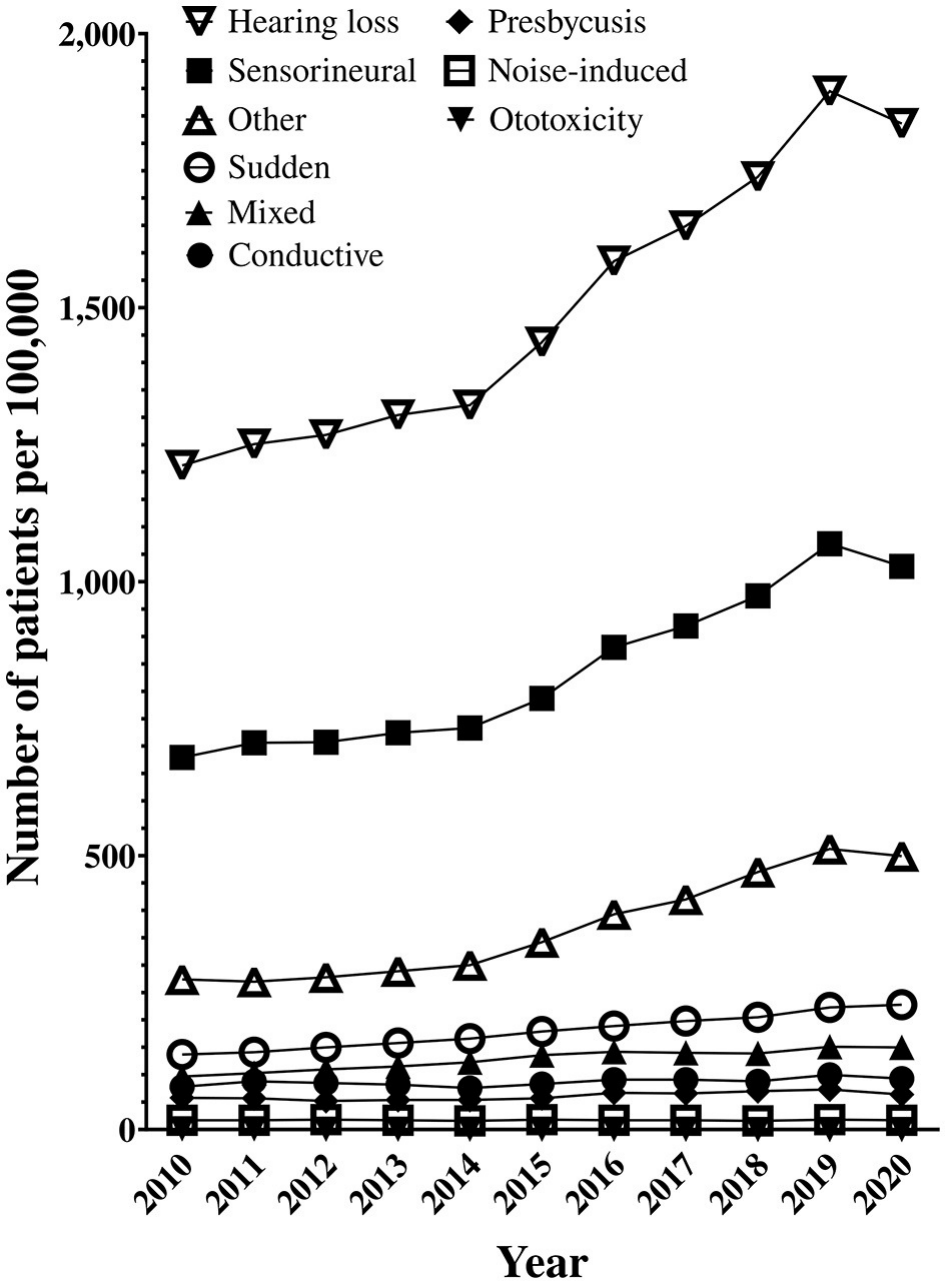
Prevalence of types of hearing loss.

When the prevalence of hearing loss was confirmed, the number of hearing loss patients by age increased from 2015 onwards (Figure 3). From 2015, the prevalence of hearing loss increased rapidly. When hearing loss was confirmed by category, “sensorineural” and “other” hearing loss accounted for the majority of hearing loss prevalence (Figure 3).

The prevalence of all types of hearing loss is increasing, especially in the conductive and other categories. The types of hearing loss were divided by age and examined according to the CAGR. Sensorineural, other, and sudden hearing loss showed a substantial increase in patients in their 20s and 30s (Table 2). In particular, the “other” hearing loss category showed a greater increase than the rest of the categories in all age groups except those under the age of 10 years. When the CAGR was calculated based on the 2015 change in hearing aid policy, the conductive and other hearing loss categories had the biggest change.

### Hearing disability and hearing aids in patients with hearing loss

The hearing aid prescription populations were compared before and after 2015. The average number of people prescribed hearing aids increased after 2015. When looking at the CAGR, the age group with the highest increase in hearing aid prescriptions was the under 10s; there was also a significant change in the teenage group. The population receiving hearing aids increased by 1.74 times in 2020 compared to 2010. This increase was 2.12 times in the total population for the same period (Table 3, Supplementary material).

**Table 3:** Hearing aid prescription and hearing disability status in South Korea.

**Number of patients for 100,000**	**2010**	**2011**	**2012**	**2013**	**2014**	**2015**	**2016**	**2017**	**2018**	**2019**	**2020**
Hearing aid	9.956	12.79	11.68	14.76	17.99	22.21	28.19	30.46	27.34	26.39	23.31
Disability	342.6	360.0	368.2	374.0	383.8	393.3	438.7	500.7	584.8	660.5	706.4
**CAGR** ^*^		**Under 10**	**10–19**	**20–29**	**30–39**	**40–49**	**50–59**	**Over 60**			
Hearing aid	2010–2014	0.486	−6.459	0.686	2.321	3.581	3.299	2.762			
	2015–2020	7.651	5.094	−1.198	0.305	−0.350	0.941	1.217			
Disability	2010–2014	−0.132	−0.087	0.258	−0.874	−2.743	−1.737	0.758			
	2015–2020	1.917	−0.171	0.465	−0.276	−0.870	−1.561	5.750			

* Compound annual growth rate.

Hearing-disabled populations were compared before and after 2015. In general, the hearing-disabled population increased from 2010 to 2020. According to the CAGR, the age group with the highest rate of increase in hearing disability is the population over 60 years. However, significant changes were also observed in those under 10 years and those in their 20s. The overall hearing-disabled population increased by 2.06 times in 2020 compared to 2010 (Table 3, Supplementary material).

We compared the hearing disability and hearing aid prescription populations before and after 2015. The hearing aid prescription population showed a clear upward trend starting in 2015. When looking at the CAGR, the increase was reflected in the under 10s population. In the case of the hearing-disabled population, there has been a clear upward trend since 2015. The age group with the greatest impact on the increase is the population over 60 years old. This is likely due to South Korea's hearing aid subsidy increase implemented in 2015. Patients who had suffered from hearing loss for an extended period had purchased a new hearing aid. Moreover, patients with hearing loss who previously could not afford a hearing aid could now afford one due to the subsidy.

### Effects of hearing disability and hearing aid prescription on the prevalence of hearing loss patients

**Figure 4:**
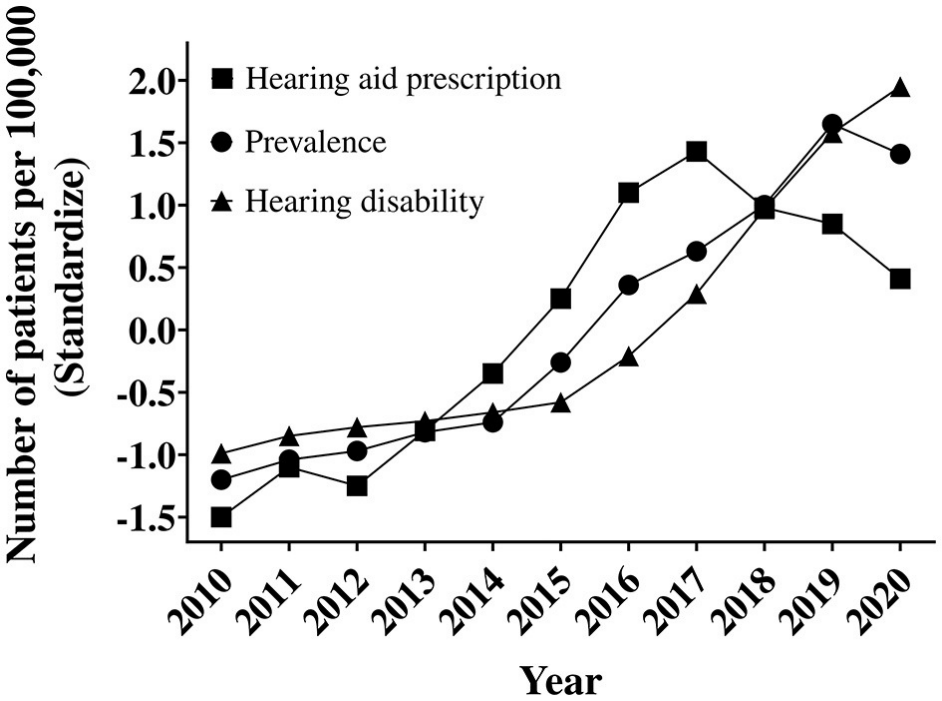
Trends in the prevalence of hearing loss, hearing aid prescription, and hearing disability.

The 2015 increase in hearing aid subsidies is expected to affect the prevalence of people prescribed hearing aids and the number of people with hearing disabilities. The increasing prevalence of hearing loss, hearing aid prescriptions, and hearing-disabled populations were compared and analyzed (Figure 4). A regression analysis was performed on the hearing aid prescription population and the increasing hearing-disabled population to establish the prevalence of hearing loss. The univariate and multivariate analyses showed that the hearing aid prescription population and the hearing disability population affected the prevalence of hearing loss. The *r*-value of each analysis was high, exceeding 0.95 (Table 4). This can be interpreted as the rising trend of hearing aid prescription adoption concurrent with the prevalence of hearing loss. The 2015 hearing aid subsidy increase affected a rapid increase in the prevalence of hearing loss.

**Table 4:** Regression analysis results of hearing disability and hearing aid prescription for the prevalence of hearing loss.

	**Univariate**			**Multivariate**		
	**β**	***p*-value**	***r***	**β**	***p*-value**	***r***
Hearing aid prescription	86.302	< 0.000	0.963	48.594	< 0.000	0.995
Hearing disability	1.847	< 0.000	0.956	0.947	< 0.000	

## Discussion

Hearing loss is a condition caused by the weakening or lack of function of the auditory organ that receives, transmits, and analyzes sounds. Hearing loss can be broadly divided into conductive and sensorineural types. The ear structure is divided into outer, middle, and inner ears. The cochlea's sensory cells and auditory nerves, which detect sound, belong to the inner ear. The outer and middle ears are responsible for efficiently transmitting surrounding sounds to the inner ear. Therefore, diseases of the inner ear cause sensorineural hearing loss, while outer or middle ear diseases cause conductive hearing loss that interferes with sound transmission (7, 19, 20).

Since a revision on 1 July 2020, there has been a change in the funding allocated to the post-management support system. However, this was not covered in this study due to the relatively short period between the revision and this study. Receiving approval for a hearing aid subsidy requires a diagnosis of hearing disability and a record of hearing aid prescriptions. This study found that this process affected the prevalence of hearing loss. There may have been patients who had previously suffered from hearing disability or had been diagnosed with hearing loss but had not been diagnosed with hearing disability. It can also be inferred that the number of people with hearing loss rapidly expanded as patients were diagnosed with hearing disabilities. These patients then received hearing aid prescriptions for eligibility for the increased hearing aid subsidy. This means that patients with undiagnosed hearing loss in South Korea were now identified. This also improved the ability to estimate the hearing loss population in South Korea more accurately. Furthermore, the pre-2015 hearing aid subsidy was less effective because the cost of diagnosis exceeded the potential hearing aid subsidy benefit (13).

However, it can be seen that the prevalence of hearing loss has declined since 2019. This was likely due to the onset of the COVID-19 pandemic and the reticence of high-risk groups (under 10s and over 60s) to expose themselves to potential infection in the overloaded medical system. However, this is not certain as it was outside the study period. If the prevalence of hearing loss continues to decrease or remains stable, these results may be interpreted differently. However, the total number of hearing loss cases increased by approximately 70% over 10 years from 1,281,169 in 2010 to 2,184,821 in 2020. The number of cases increased by 8% in 2015 and 12% in 2016, and the rate of increase in 2013 and 2014 was approximately 3%.

South Korea's NHI system is operated by a single insurance company, the NHIS, per the National Health Insurance Act of 1963. The NHIS DB used in this study was obtained from South Korea's NHI system. South Korea's medical infrastructure and NHI provide some of the best coverage globally, and patient reluctance to diagnose and treat is very low (21). Therefore, the quality of the NHIS DB obtained from the NHI is very reliable. Research using the NHI database system has been published on various topics related to healthcare, health policy, and diseases such as cancer, infection, heart disease, cerebrovascular disease, hypertension, diabetes, and endocrine diseases. Additionally, the credibility of the database can be demonstrated in a study published through the COVID-19 International Joint Research Project using 2020 NHI national data comparing COVID-19 and influenza patients (10, 22–27). Moreover, the present study was conducted through a memorandum of understanding (MOU) with the NHIS at the Korea Hearing Big Data Center (KHBC); the cooperation and consultation of the NHIS were recognized.

The KHBC was established in 2020 as part of South Korea's big data platform business as an affiliate of the Korean Audiological Society. The purpose of the KHBC was to provide specialized big data on hearing to medical device industries and researchers. As of 2022, the KHBC has approximately 1,00,000 data points, including reference standard data for the hearing threshold of Koreans from the Korea Hearing Standard Reference Data Center. We are researching hearing big data through the MOU with NHIS. We are also exploring the combination of NHIS DB and KHBC data. This is the first MOU study conducted by the KHBC with the NHIS.

### Limitations

This study has the following limitations. After the increase in the prevalence of hearing loss, it was not possible to confirm the stabilization, increase, or decrease in the prevalence of hearing loss. Furthermore, a study on post-subsidy supports that funding in July 2020 was not considered due to the short study period. Our study is not perfect in the operational definition as it thoroughly relied on the ICD-10 by the doctor when the patient visits the hospital. The solution to this is to add different definition conditions for each type of hearing loss, depending on the various causes. However, the ICD-10's other hearing loss is not well defined. Therefore, the prevalence rates in our study are estimates. The present study also covered the period during the onset of the COVID-19 pandemic, which may impact the generalizability of the study results. The study period should be extended to investigate the cause of the changes in the prevalence of hearing loss after the study period.

### Conclusion

In South Korea, it is necessary to undergo a process to receive a hearing aid subsidy. This includes the diagnosis of a hearing disability and being prescribed a hearing aid. This process affects the prevalence of hearing loss. Therefore, the 2015 increase in hearing aid subsidies impacted the prevalence of hearing loss in the medium and short term. Based on the category of hearing loss, a surge in sensorineural hearing loss and other types of hearing loss was observed after the increased hearing aid subsidies. The prevalence of hearing loss was, therefore, affected by the increase in the hearing-disabled population and hearing aid adoption due to changes in the hearing aid subsidy. Caution is needed when studying the period in the mid-long term.

## Data availability statement

The data analyzed in this study was obtained from the Korean National Health Insurance Service (NHIS) the following licenses/restrictions apply: only Korean researchers can access these datasets. Requests to access these datasets should be directed to NHIS, https://nhiss.nhis.or.kr/bd/ab/bdaba000eng.do.

## Ethics statement

The studies involving humans were approved by the Institutional Review Board (IRB) of Yonsei University Wonju Severance Christian Hospital (CR321338). The studies were conducted in accordance with the local legislation and institutional requirements. The Ethics Committee/Institutional Review Board waived the requirement of written informed consent for participation from the participants or the participants' legal guardians/next of kin because this study is a study using data collected retrospectively and the data collected is not data collected for research.

## Author contributions

YS: conceptualization. CY and JL: data curation and visualization. CY: formal analysis, methodology, and writing—original draft. CY, YS, and TK: project administration and writing—review and editing. All authors contributed to the article and approved the submitted version.
