# Supplementary material for: Effect of changes in the hearing aid subsidy on the prevalence of hearing loss in South Korea
Source: Front Neurol. 2023 Sep 12;14:1215494. doi: 10.3389/fneur.2023.1215494 (PMC10536239; doi:10.3389/fneur.2023.1215494)
Supplement: Supplementary file 1 [file Table_1.pdf]

Supplement

Prevalence by hearing loss category (number of patients per 100,000 population), annual growth rate by hearing aid prescription & hearing disability

Type of HL	SEX	AGE	2010	2011	2012	2013	2014	2015	2016	2017	2018	2019	2020
Conductive	M	Under 10	90	111	97	88	75	76	83	73	66	102	67
		10–19	76	94	85	77	69	72	80	82	73	81	68
		20–29	49	60	61	61	56	64	68	72	66	70	68
		30–39	52	59	60	57	56	62	65	68	66	67	64
		40–49	57	62	61	60	56	61	65	65	62	65	63
		50–59	76	80	79	75	70	77	81	79	79	89	83
		Over 60	108	109	106	103	94	107	121	121	122	137	130
	F	Under 10	79	89	79	76	62	62	68	61	54	84	54
		10–19	69	81	75	74	66	70	80	77	67	73	66
		20–29	69	86	88	89	82	92	107	105	94	97	95
		30–39	70	82	84	80	72	78	91	91	88	94	91
		40–49	80	87	86	82	77	83	90	87	86	92	88
		50–59	105	115	116	111	101	111	117	122	117	133	120
		Over 60	119	116	115	114	107	119	127	128	128	151	138
Sensorineural	M	Under 10	246	257	254	251	246	222	234	234	226	237	186
		10–19	272	304	294	289	298	288	309	322	310	317	305
		20–29	280	317	309	318	326	328	342	367	360	385	397
		30–39	321	326	337	348	352	353	382	399	397	453	454
		40–49	446	451	444	441	447	451	480	492	487	529	531
		50–59	827	813	787	769	754	792	825	827	844	887	858
		Over 60	2,115	1,975	2,034	2,045	1,986	2,185	2,518	2,537	2,686	2,874	2,627
	F	Under 10	205	211	199	201	202	172	191	190	183	198	162
		10–19	260	294	301	315	319	316	343	355	328	355	363
		20–29	339	387	400	426	445	459	496	539	541	564	616
		30–39	384	409	424	431	449	461	507	537	565	623	656
		40–49	516	536	521	529	533	557	590	595	602	666	681
		50–59	961	972	924	911	902	952	987	982	1,011	1,060	1,009
		Over 60	2,061	2,010	2,038	2,048	2,028	2,176	2,409	2,473	2,610	2,788	2,462
Mixed	M	Under 10	39	44	41	48	55	50	41	33	30	37	26
		10–19	48	58	58	58	62	64	66	67	58	63	55
		20–29	43	50	55	55	62	68	70	69	66	71	71
		30–39	51	55	62	66	70	77	80	82	78	83	86
		40–49	68	69	74	77	80	87	91	86	86	89	91
		50–59	113	111	116	118	121	133	134	125	122	129	127

		Over 60	253	229	249	257	264	302	326	310	310	322	307
	F	Under 10	34	37	36	40	48	46	33	28	24	31	22
		10–19	44	60	63	67	68	74	70	72	66	69	69
		20–29	65	75	82	89	97	106	114	111	112	114	120
		30–39	73	79	91	91	105	109	113	111	113	124	130
		40–49	89	94	97	99	106	114	116	113	110	122	125
		50–59	149	152	162	159	170	176	174	165	158	174	167
		Over 60	256	248	269	278	286	315	331	327	323	343	322
**Ototoxicity**	M	Under 10	1.79	1.80	0.54	0.42	0.34	0.34	0.25	0.26	0.36	0.23	0.10
		10–19	1.09	0.79	0.38	0.24	0.41	0.26	0.34	0.18	0.18	0.11	0.16
		20–29	0.84	0.54	0.20	0.20	0.26	0.23	0.23	0.14	0.14	0.06	0.14
		30–39	0.54	0.79	0.31	0.17	0.20	0.20	0.05	0.05	0.03	0.08	0.03
		40–49	0.97	0.92	0.43	0.52	0.34	0.43	0.36	0.25	0.23	0.19	0.19
		50–59	1.96	1.98	0.87	0.43	0.60	0.49	0.46	0.64	0.35	0.30	0.14
		Over 60	3.92	4.47	1.77	1.68	1.61	1.54	0.95	1.08	1.16	1.00	0.89
	F	Under 10	1.42	1.41	0.40	0.18	0.22	0.13	0.27	0.23	0.33	0.15	0.05
		10–19	0.50	0.72	0.39	0.33	0.34	0.18	0.19	0.15	0.16	0.12	0.13
		20–29	0.54	0.68	0.25	0.25	0.32	0.29	0.19	0.28	0.12	0.12	0.03
		30–39	0.59	0.67	0.45	0.28	0.18	0.11	0.14	0.11	0.08	0.09	0.03
		40–49	0.84	1.01	0.61	0.44	0.48	0.35	0.23	0.09	0.12	0.22	0.12
		50–59	1.79	1.93	0.98	0.67	0.75	0.32	0.39	0.46	0.45	0.40	0.28
		Over 60	2.88	3.14	1.50	1.61	1.45	1.77	1.05	0.95	0.71	0.74	0.49
**Presbycusis**	M	Under 10	0	0	0	0	0	0	0	0	0.04	0	0
		10–19	0.03	0	0	0	0	0	0	0	0	0	0
		20–29	0	0	0	0	0	0.03	0.11	0	0	0	0
		30–39	0	0	0	0	0	0	0	0.03	0.03	0.03	0
		40–49	2.19	1.53	2.19	1.55	1.92	1.61	1.92	1.44	1.39	1.35	1.19
		50–59	20	20	17	16	15	15	16	14	14	14	11
		Over 60	350	307	283	288	272	286	326	307	312	313	259
	F	Under 10	0	0	0	0	0	0	0	0	0	0	0
		10–19	0.06	0	0	0	0	0	0	0	0	0	0
		20–29	0.06	0	0	0	0	0	0	0	0	0	0
		30–39	0	0	0	0	0	0	0.03	0	0	0.06	0
		40–49	2.01	1.40	1.68	1.53	1.52	1.41	2.14	1.43	1.15	1.44	1.31
		50–59	20	21	17	17	17	16	17	13	12	13	10
		Over 60	375	351	321	320	312	310	344	323	329	328	269
**Sudden**	M	Under 10	14	12	13	16	16	13	11	9	10	15	11
		10–19	53	58	57	59	61	63	67	71	65	67	67

		20–29	78	86	90	99	100	106	111	118	114	127	137
		30–39	103	109	117	125	130	138	143	155	154	167	177
		40–49	135	141	145	148	160	170	180	188	192	210	212
		50–59	199	194	205	211	220	232	239	244	247	263	257
		Over 60	264	252	267	282	288	309	322	328	341	361	349
	F	Under 10	12	10	12	13	14	11	11	10	9	13	10
		10–19	59	63	69	73	76	81	83	82	78	80	92
		20–29	109	126	134	144	147	156	171	180	179	187	217
		30–39	137	146	154	158	166	183	196	207	218	231	257
		40–49	166	173	184	194	201	216	227	238	254	274	284
		50–59	234	237	251	253	267	281	282	295	308	333	329
		Over 60	257	235	257	266	272	296	312	314	322	349	333
**Noise-induced**	M	Under 10	0.92	1.48	1.29	1.42	1.18	1.35	1.27	1.30	1.25	0.92	0.43
		10–19	8	11	9	7	8	9	8	9	7	8	6
		20–29	25	27	25	25	22	23	20	20	18	17	14
		30–39	20	19	19	17	17	17	15	16	14	15	12
		40–49	28	27	28	26	25	27	26	26	23	24	22
		50–59	44	42	44	40	41	44	39	39	41	41	39
		Over 60	30	30	35	34	34	39	39	36	43	52	56
	F	Under 10	0.73	1.01	1.33	1.02	0.94	0.94	0.99	1.01	0.33	0.97	0.40
		10–19	8	9	9	7	8	8	7	8	5	6	5
		20–29	10	10	12	12	10	12	11	11	8	7	7
		30–39	9	8	8	7	7	7	7	7	6	5	5
		40–49	12	13	12	11	10	11	9	10	7	8	7
		50–59	16	17	17	16	15	18	16	15	14	13	12
		Over 60	12	12	13	12	12	15	13	12	12	14	14
**Other**	M	Under 10	231	232	242	240	215	230	229	228	242	267	207
		10–19	150	161	157	158	158	176	198	215	223	231	205
		20–29	131	137	140	142	152	170	189	206	220	221	239
		30–39	132	136	145	157	168	195	213	225	243	266	267
		40–49	182	175	181	189	202	223	241	263	275	303	308
		50–59	321	300	298	309	316	346	381	391	427	447	445
		Over 60	707	599	616	635	638	738	871	890	1,018	1,111	1,029
	F	Under 10	189	188	199	195	177	174	190	183	197	214	164
		10–19	134	152	157	161	164	175	208	224	230	235	240
		20–29	152	173	188	199	220	247	282	312	332	334	366
		30–39	170	184	199	206	234	266	301	329	361	385	400
		40–49	230	227	239	249	257	291	327	349	379	402	413

	50–59	418	390	396	395	397	443	487	516	559	584	563
	Over 60	672	608	624	635	655	738	864	908	1,028	1,116	1,014
**Hearing aid prescription**			**2011**	**2012**	**2013**	**2014**	**2015**	**2016**	**2017**	**2018**	**2019**	**2020**
**ACR***	Under 10		-4.39	-20.73	22.55	12.47	-18.19	36.30	32.46	25.17	-8.67	-5.93
	10–19		-42.32	11.72	8.68	-21.71	29.17	22.50	18.15	26.05	-31.91	25.91
	20–29		1.37	11.82	9.10	-14.01	1.31	1.25	29.86	3.43	-24.25	-12.91
	30–39		34.30	-29.48	36.26	-4.73	11.27	15.43	-15.97	10.68	-1.66	-2.64
	40–49		32.60	-8.07	8.18	4.07	9.80	13.21	-6.88	-0.67	-6.83	-0.69
	50–59		15.04	-16.24	16.11	19.71	2.54	17.72	4.32	-12.47	0.04	1.18
	Over 60		9.19	-8.68	13.34	13.08	17.00	22.04	1.86	-3.42	2.41	-9.32
**Hearing disability**			**2011**	**2012**	**2013**	**2014**	**2015**	**2016**	**2017**	**2018**	**2019**	**2020**
**ACR***	Under 10		-0.51	-2.04	-1.27	2.70	2.18	6.50	4.11	5.46	2.53	-1.05
	10–19		-0.91	1.36	-1.01	-0.20	-0.83	-0.31	0.17	-0.70	-0.23	-0.47
	20–29		0.78	-0.84	1.45	0.95	1.92	0.97	1.22	1.52	0.02	0.47
	30–39		-1.62	-2.03	-3.22	-0.94	-1.25	-0.26	0.64	-0.71	-0.08	-2.05
	40–49		-6.31	-6.70	-5.17	-6.08	-5.60	-1.30	-2.60	-1.41	-0.98	-1.50
	50–59		-2.51	-6.01	-3.60	-3.31	-4.45	-0.84	-1.89	-0.66	-4.02	-6.44
	Over 60		5.20	1.45	-0.50	0.79	0.04	10.57	13.40	15.49	10.55	3.32

***** Annual growth rate
